# Potential mesenchymal stem cell therapeutics for treating primary biliary cholangitis: advances, challenges, and perspectives

**DOI:** 10.3389/fcell.2022.933565

**Published:** 2022-07-18

**Authors:** Yanlei Yang, Robert Chunhua Zhao, Fengchun Zhang

**Affiliations:** ^1^ Clinical Biobank, National Science and Technology Key Infrastructure on Translational Medicine in Peking Union Medical College Hospital, Medical Science Research Centre, Medical Science Research Centre, Peking Union Medical College Hospital, Chinese Academy of Medical Sciences and Peking Union Medical College, Beijing, China; ^2^ The Ministry of Education Key Laboratory, Department of Rheumatology and Clinical Immunology, Peking Union Medical College Hospital, Clinical Immunology Center, Chinese Academy of Medical Sciences and Peking Union Medical College, Beijing, China; ^3^ Beijing Key Laboratory, Institute of Basic Medical Sciences Chinese Academy of Medical Sciences, School of Basic Medicine, Peking Union Medical College, Peking Union Medical College Hospital, Center of Excellence in Tissue Engineering, Chinese Academy of Medical Sciences, Beijing, China; ^4^ School of Life Sciences, Shanghai University, Shanghai, China

**Keywords:** mesenchymal stem cells (MSCs), cell based–therapy, primary biliary cholangitis (PBC), immunomodulaiton, exosome

## Abstract

Primary biliary cholangitis (PBC) is a cholestatic autoimmune liver disease characterized by the gradual destruction of small intrahepatic bile ducts that eventually leads to liver cirrhosis, failure, and even carcinoma. The treatment options for PBC are limited, and the main treatment choices are the US Food and Drug Administration–approved ursodeoxycholic acid and obeticholic acid. However, many patients fail to respond adequately to these drugs and the adverse effects frequently lead to low life quality. For patients with end-stage PBC, liver transplantation remains the only effective treatment. Given their low immunogenicity, prominent immunomodulation property, differentiation potential, and tissue maintenance capacity, mesenchymal stem cells (MSCs) are emerging as new options for treating liver diseases, including PBC. Accumulating evidence from basic research to clinical studies supports the positive effects of MSC-based therapy for treating PBC. In this review, we characterized the underlying roles and mechanisms of MSCs for treating liver diseases and highlight recent basic and clinical advances in MSC-based therapy for treating PBC. Finally, the current challenges and perspectives for MSC-based therapy in clinical application are discussed, which could help accelerate the application of MSCs in clinical practice, especially for refractory diseases such as PBC.

## Introduction

Primary biliary cholangitis (PBC), which was previously termed as primary biliary cirrhosis, is a chronic cholestatic autoimmune liver disease (Beuers et al., 2015a; Lleo et al., 2020). The continuous autoimmune stimuli cause selective destruction of the small and medium intrahepatic bile ducts, leading to intrahepatic cholestasis that induces ductular proliferation, which subsequently contributes to cholangiocyte death, liver fibrosis, cirrhosis, liver failure, and even hepatocellular carcinoma (Lindor et al., 2009; Carey et al., 2015; Rodrigues et al., 2018; Lleo et al., 2020). PBC is a familiar autoimmune-related liver disease with an overall prevalence of 118.75 cases per million people in the Asia-Pacific region (Zeng et al., 2019). Middle-aged women are most affected by PBC, with a female: male ratio of 10:1.6 (Invernizzi et al., 2004; Lleo et al., 2016; Rodrigues et al., 2018). The hallmarks of PBC diagnosis are the serum autoimmune antibodies, including anti-mitochondrial antibodies (AMAs, >95% positive in PBC patients) that target the pyruvate dehydrogenase complex E2 subunit (PDC-E2), and anti-nuclear antibodies (ANAs), the 210-kDa glycoprotein of the nuclear pore complex (anti-gp210), and the nuclear antigen Sp100 (anti-sp100) (Courvalin et al., 1990; Sternsdorf et al., 1995; Hu et al., 2014; Rodrigues et al., 2018). Furthermore, obscure chronic elevation of alkaline phosphatase (ALP) combined with an AMA titer exceeding 1:40 can also be diagnosed as PBC (European Association for the Study of the Liver, 2017).

**FIGURE 1:**
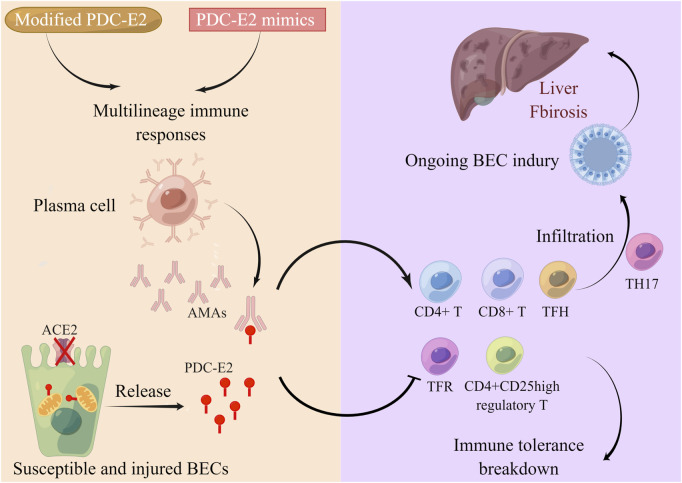
Systemic autoimmune response in PBC pathogenesis. The main AMA targeting PBC-E2 is expressed on the inner mitochondrial membrane. When exposed to environmental PDC-E2 mimic or modified PDC-E2, multilineage immune responses are triggered to attack the BECs. Plasma cells then generate disease-specific AMAs to target immunodominant PDC-E2 epitopes on the BECs, causing BEC injury. AE2 is localized on the apical domain of BECs. BECs with dysfunctional AE2 are susceptible to apoptosis, which further exposes PDC-E2 to circulating AMAs, resulting in extensive cellular injury. Autoreactivity-suppressing CD4^+^CD25^high^ Tregs and T follicular regulatory (TFR) cells are downregulated in PBC, accounting for the disruption of immune tolerance. Proinflammatory effector CD4^+^ and CD8^+^ T cells and TFH cells infiltrate the portal tracts, while Th17 infiltration is also observed in PBC targeting damaged cholangiocytes, which leads to the advanced fibrosis stage of PBC.

PBC is a multifactor polygenic disease. Genetic susceptibility (Selmi et al., 2004), immune tolerance breakdown, epigenetic modification (Rodrigues et al., 2018), and environmental triggers (Selmi and Gershwin, 2009) collaborate to contribute to PBC occurrence and progression (Gulamhusein and Hirschfield, 2020). The core feature of PBC is the systemic autoimmune response, which causes progressive lymphocytic cholangitis (Figure 1). The main AMA target PBC-E2 is expressed on the inner mitochondrial membrane. Following exposure to environmental PDC-E2 mimics or modified PDC-E2, multilineage immune responses are triggered to attack biliary epithelial cells (BECs) (Yeaman et al., 1988; Gulamhusein and Hirschfield, 2020). Plasma cells then generate disease-specific AMAs to target immunodominant PDC-E2 epitopes on the BECs, causing BEC injury. Localized on the apical domain of BECs, anion exchanger 2 (AE2) is necessary for maintaining the biliary HCO3^−^ umbrella and protecting cholangiocytes from apoptosis (Hohenester et al., 2012; Rodrigues et al., 2018). BECs with dysfunctional AE2 are susceptible to apoptosis, which further exposes PDC-E2 to circulating AMAs, resulting in extensive cellular injury (Gulamhusein and Hirschfield, 2020). The vital role of the adaptive immune system in PBC pathogenesis is well recognized. CD4^+^ and CD8^+^ T cells and T follicular helper (TFH) cells are proinflammatory effector T cells that exhibit antigen-specific infiltration in the portal tracts (Kita et al., 2002; Shimoda et al., 2008). CD4^+^CD25^high^ regulatory T cells (Tregs) and T follicular regulatory cells are autoreactivity-suppressing cells that are downregulated in PBC, accounting for the disruption of immune tolerance (Lan et al., 2006; Zheng et al., 2017). T helper 17 (Th17) cell infiltration is also observed in PBC, accompanied by increased interleukin IL-6, IL-17, and transforming growth factor-β1 (TGF-β) cytokines targeting damaged cholangiocytes, leading PBC to an advanced fibrosis stage (Rong et al., 2009; Yang et al., 2014; Gulamhusein and Hirschfield, 2020).

The traditional treatment is mainly based on bile acid drugs, including ursodeoxycholic acid (UDCA), which alleviates cholestasis, inflammation, and fibrogenesis (Beuers et al., 2015b). Guidelines suggest UDCA as the first-line treatment for patients diagnosed with PBC (Lindor et al., 2019). However, UDCA is only effective in early-stage patients for delaying PBC progression; approximately 25%–50% of PBC patients do not respond to UDCA (Gong et al., 2007; Shah and Kowdley, 2020; He et al., 2021). The addition of obeticholic acid (OCA) as a second-line drug is suggested for such patients, but this treatment is usually terminated due to adverse effects such as pruritus, which occur in 10% of the patients (Beuers et al., 2015b; Nevens et al., 2016). Moreover, liver transplantation is the only effective cure for end-stage PBC, but is limited by the shortage of liver donors, requirement for lifelong immunosuppression, and financial considerations (Arsenijevic et al., 2017; Melchor-Mendoza et al., 2017). Therefore, there is an urgent need to explore new treatment options. Currently, mesenchymal stem cell (MSC)–based therapy is emerging as a new alternative treatment for PBC patients, as MSCs have low immunogenicity and prominent immunomodulation property, differentiation potential, and tissue maintenance capacity. In this review, we characterized the underlying roles and mechanisms of MSCs in treating liver diseases, and then highlight recent basic and clinical advances in MSC-based therapy for treating PBC. Finally, we discussed the current challenges and perspectives for MSC-based therapy in clinical application, which could help accelerate the clinical practice of MSC, especially for refractory diseases such as PBC.

## Underlying roles and mechanisms of mesenchymal stem cells in treating the liver disease

**FIGURE 2:**
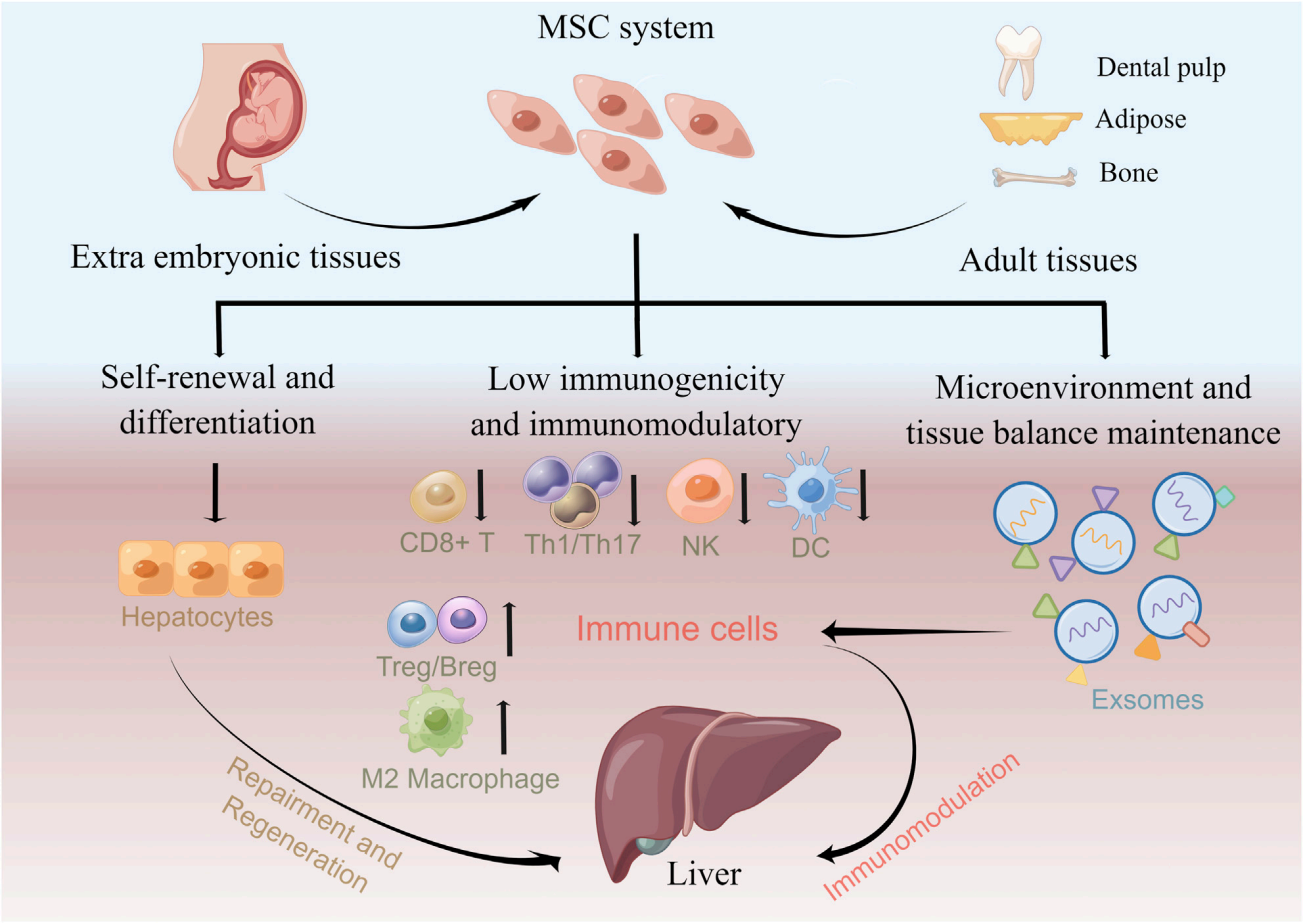
Underlying roles of MSCs in treating liver diseases. MSCs can be isolated from a variety of tissues that constitute MSC systems. MSCs contribute to clinical efficacy in liver diseases *via* hepatocyte differentiation potential and immunomodulation function. MSCs can reconstitute liver function *in vivo* by differentiating into hepatocytes. Furthermore, MSCs modulate the immune response and attenuate liver disease by increasing autoreactivity, suppressing Tregs/Bregs and anti-inflammatory M2 macrophages, and suppressing CD8^+^ T, Th1/Th17, NK cell, and DC immune responses. MSC exosomes also exhibit immunomodulation effects by affecting the immune cell response.

MSCs are multipotent mesoderm-derived adult stem cells with a broad distribution of sources and low immunogenicity and immunomodulatory function (Zhao, 2013; Wang et al., 2019). They are attractive choices for cell therapy that has been used for treating hematological diseases, autoimmune diseases, peripheral nerve injuries, and COVID-19 (Chen et al., 2019b; Yousefi et al., 2019; Leng et al., 2020; Zoehler et al., 2020; Zhu et al., 2021). However, the detailed mechanism underlying MSC-based therapy is not fully understood. Based on current studies, MSCs contribute to clinical efficacy for the liver disease in the following ways: hepatocyte differentiation potential and immunomodulatory function (Figure 2).

In hepatic injury mouse models, MSCs can reconstitute liver function *in vivo* by differentiating into hepatocytes (Banas et al., 2007; Aurich et al., 2009; Xu et al., 2014; Fu et al., 2016). Furthermore, MSC administration routes can influence their homing and subsequent differentiation; compared with intraperitoneal injection, intrahepatic MSC injection develops more efficient hepatocytes (Chamberlain et al., 2007). However, accumulating clinical applications indicate that only a small fraction of MSCs undergo differentiation while still yielding effective results (Ferrand et al., 2011; Lai et al., 2015; Vizoso et al., 2019), indicating that MSC-mediated immunomodulation through the secretion of bioactive factors and contact with immune cells may confer MSC efficacy (Gao et al., 2016).

MSCs secrete multiple bioactive factors and extracellular vesicles (EVs), which constitute the MSC secretome and contribute to immunomodulation, tissue development, cell differentiation, and hematopoietic support (Lai et al., 2015; Konala et al., 2016; Yang et al., 2021). Accumulating evidence indicates that MSCs exert their immunomodulatory function in the liver through the secretome and direct cell–cell contact with immune cells such as T cells, B cells, macrophages, natural killer (NK) cells, dendritic cells (DCs), and Tregs.

Comprising the majority of liver immune cells, macrophages can be classified as proinflammatory M1 and anti-inflammatory M2 types (Italiani and Boraschi, 2014; Wen et al., 2021). MSCs hold the potential for regulating macrophage polarization and promoting M2 differentiation both *in vivo* and *in vitro*. In an ischemia–reperfusion (IR)–induced liver sterile inflammatory injury mouse model, MSC infusion reprogrammed macrophage polarization from the M1 to M2 phenotype by activating the macrophage Hippo–YAP–β-catenin–NLRP3 pathway, thereby reducing hepatocellular damage (Li et al., 2019). Kim and Hematti (2009) indicated that macrophages can be educated to an anti-inflammatory state (MSC-educated macrophages, MEM) expressing high IL-10 and IL-6 and low IL-12 and TNF-α levels *in vitro* (Kim and Hematti, 2009). Their group further demonstrated that MEMs express high IL-6 and are protective in lethal graft-versus-host disease (GVHD) and radiation injury models (Bouchlaka et al., 2017).

MSCs secrete anti-inflammatory factors to inhibit T-cell proliferation, including IL-10, IL-6, TGF-β, nitric oxide, prostaglandin E2 (PGE2), indoleamine 2,3-dioxygenase (IDO), hepatocyte growth factor, programmed cell death 1 ligand 1 (PD-L1), heme oxygenase-1 (HO-1), and galectins. Moreover, MSCs can secrete MCP-1 to induce Fas-mediated T-cell apoptosis (Akiyama et al., 2012). Furthermore, IL-10 produced by MSCs hinders naïve CD4^+^ T-cell differentiation to proinflammatory Th1 and Th17 cells by increasing the proportion of CD4^+^CD25^+^Foxp3^+^ Tregs (Luz-Crawford et al., 2013). MSC-secreted TGF-β promotes Treg differentiation by activating the Smad2 pathway (Tang et al., 2015). MSCs can also inhibit B-cell proliferation and their immunoglobulin (IgG1) and IgM production (Corcione et al., 2006; Asari et al., 2009; Rosado et al., 2015). In addition, MSCs can induce CD19^+^CD24^high^CD38^high^ and CD23^+^CD43^+^ regulatory B cells (Bregs) in inflammatory bowel disease, producing more IL-10 to promote B-cell immunosuppressive properties (Franquesa et al., 2015; Chen et al., 2019a).

NK cells are crucial players in innate immunity. MSC-secreted IDO and PGE2 can strongly inhibit NK cell proliferation, cytotoxicity, and cytokine production (Spaggiari et al., 2008; Hu et al., 2019). A study using a liver injury model demonstrated that MSCs suppress liver NK cell recruitment and activation (Qu et al., 2015). DCs are pivotal antigen-presenting cells and MSCs can suppress their activation, maturation, and migration (Gao et al., 2017). Notably, MSC administration in a liver injury model promoted the differentiation of CD11c^+^B220^−^ DC precursors into regulatory DCs (Zhang et al., 2014).

In recent years, MSC exosomes have been increasingly viewed as potential therapeutic approaches and exhibit immunomodulation effects. For example, MSC exosomes inhibited T- and B-cell proliferation and decreased B-cell IgM production (Khare et al., 2018). TNF-α treatment stimulated increased exosome secretion by gingival tissue–derived MSCs and enhanced the exosomal CD73 expression, thereby promoting anti-inflammatory M2 macrophage polarization (Nakao et al., 2021). Moreover, MSCs released small EVs (sEVs) *in vivo* that targeted M2 macrophages and increased TGF-β levels, subsequently alleviating severe spinal cord injury in a rodent model (Nakazaki et al., 2021).

MSCs exert multi-faceted immunomodulatory effects by secreting biofactors and interacting with immune cells. However, the effects may vary in specific diseases and depend on their received types of inflammatory signals.

## Therapeutic potential and mechanism of mesenchymal stem cells in treating primary biliary cholangitis

### Basic research on mesenchymal stem cells for treating primary biliary cholangitis

In 2011, Wang et al. (2011) constructed a mouse model using polyinosinic–polycytidylic acid sodium (polyI: C), mimicking the PBC disease phenotype to explore the effect of allogeneic bone marrow–derived MSCs (BMSCs) on the model (Wang et al., 2011). The mice exposed to 16 consecutive weeks of polyI: C administration showed increased serum ALP, AMA, and ANA levels, mononuclear cell infiltration around the bile ducts, and decreased CD4^+^Foxp3^+^ Tregs in the spleen and mesenteric lymph nodes. After 6 weeks of BMSC transplantation, the BMSC-transplanted mice demonstrated decreased serum ALP, AMA titers, and the inflammatory cytokine IFN-γ and had ameliorated monocyte infiltration around the bile ducts, which indicated that BMSC transplantation may attenuate liver injury mediated by the Th1 immune response. In addition, the BMSCs increased the frequency of peripheral and lymph node Tregs and serum TGF-β1, which can promote Treg differentiation. Notably, MSCs can also secrete TGF-β1 to exert their immunomodulatory effects by promoting Treg generation (Patel et al., 2010); therefore, the upregulation of Tregs in BMSC-transplanted mice may account for MSC transplantation.

Fan et al. (2018) established an autoimmune cholangitis mouse model using 2-octynoic acid coupled to bovine serum albumin (2OA-BSA) to examine the curative effect and potential mechanism of umbilical cord–derived MSCs (UC-MSCs) in treating PBC (Fan et al., 2018). They found that UC-MSC transplantation alleviated the inflammatory response and bile duct injury caused by 2OA-BSA in the liver, with decreased ALT (alanine aminotransferase), AST (aspartate aminotransferase), ALP, GGT (γ-glutamyltransferase), and serum anti-PDC-E2 autoantibodies. Moreover, no cross-species immunoreaction was observed in C57BL/6 mice that received human UC-MSCs. UC-MSC transplantation attenuated aberrant Th1 and Th17 responses by downregulating IFN-γ^+^CD4^+^ Th1 and IL-17A^+^CD4^+^ Th17 cells in the liver, spleen, and blood and downregulated liver IFN-γ, IL-12, IL-17A, and IL-23 mRNA levels. Furthermore, high Gal-9 expression in MSCs is dispensable for suppressing CD4^+^ T-cell proliferation and regulating Th1 and Th17 cell differentiation, which may be mediated by the STAT and JNK pathways.

The animal models yielded promising results for the therapeutic advances in treating PBC with allogeneic MSCs and can guide their clinical application. Nevertheless, there is an urgent need for extensive studies to clarify the underlying therapeutic mechanisms.

Cholangiocyte damage and senescence are crucial pathogenic processes in PBC. Recently, Chen et al. (2021) established an *in vitro* PBC model by generating organoids (cholangioids) from mouse liver duct–derived cholangiocytes and induced cholangioid senescence with persistent oxidative stress (H_2_O_2_). They reported that exosomes derived from human placental MSCs delayed the progression of senescence and exerted a protective effect on the cholangioids by downregulating the cell cycle arrest proteins p16^INK4A^ and p21^WAF1/Cip1^ and decreased senescence-associated secretory phenotype (SASP) components and chemokines (Chen et al., 2021). They presented a new *in vitro* cell model for the pathogenesis and mechanism study of liver diseases, including PBC, which may also be useful for developing novel drugs and therapy.

### Clinical trials of mesenchymal stem cells for treating primary biliary cholangitis

There are currently three clinical trials registered for MSC-based therapy for PBC (ClinicalTrials.gov). Wang et al. (2013) reported a pilot study for treating PBC patients who responded incompletely to UDCA with UC-MSCs (ClinicalTrials.gov Identifier: NCT01662973) (Wang et al., 2013). Seven patients were enrolled and intravenously infused with 0.5 × 10^6^ cells/kg UC-MSCs once every 4 weeks on three occasions in combination with traditional UDCA treatment. After 48 weeks of follow-up, significant alleviation of common PBC symptoms such as fatigue and pruritus, and decreased serum ALP and GGT levels were observed, with no obvious adverse effects or long-term complications. The study validated the idea that UC-MSCs are safe and feasible for treating PBC and yielded promising results for MSC therapy in other diseases.

Immediately following this study, our group conducted a study that involved 10 UDCA-resistant PBC patients, treating them with allogeneic BMSCs (ClinicalTrials.gov Identifier: NCT01440309) (Wang et al., 2014). The BM-MSCs were derived from the patients’ healthy first-degree relatives and were intravenously infused into the patients at 3–5 × 10^5^ cells/kg. No adverse events were observed after BMSC infusion and during the follow-up period. The patients who received BMSCs demonstrated improved life quality, including pruritus, fatigue, and emotional function and decreased serum ALT, AST, GGT, DBIL (direct bilirubin), and IgM levels. More importantly, the immunomodulatory effect of BM-MSCs may contribute to the treatment effectiveness of patients, including increasing CD4^+^CD25^+^Foxp3^+^ T cells and IL-10 levels and reducing CD8^+^ T cells. We also indicated that the effect of MSC infusion may be maintained for 12 months and may be optimal at 3–6 months.

In 2018, Han et al. initiated the third clinical trial for treating PBC with MSCs (ClinicalTrials.gov Identifier: NCT03668145). They aimed to enroll 140 participants to investigate the safety and efficacy of MSCs in UDCA-resistant PBC patients. Patients were randomly assigned to receive MSCs (0.1–1 × 10^6^ cells/kg *via* the peripheral vein, once in 4 weeks, three times) and UDCA, or UDCA alone. The patients’ serum ALP levels were tested at entry and 1, 3, 6, and 24 months after infusion to measure the primary outcome. Improvement of symptoms in liver histology and other liver function indices such as ALT, total bilirubin, AST, and GGT were analyzed 6 months after infusion to measure the secondary outcomes. This is an ongoing trial and its detailed data and results may be published soon.

To date, only two studies have reported the safety and efficacy of the clinical MSC application for treating PBC patients (Wang et al., 2013; Wang et al., 2014). However, both studies enrolled a small sample size, which is their main limitation. Randomized larger-scale studies and intensive mechanistic exploration of the therapeutic effect of MSCs in PBC are necessary for future clinical trials.

## Challenges and perspectives

MSCs hold great promise for treating immune disorder–related diseases. However, global clinical trials for MSC-based PBC treatment are relatively rare and progress slowly. On the one hand, there are concerns about the clinical application of MSCs. The first of these involves safety. A recent retrospective meta-analysis of 62 randomized clinical trials in the past 15 years indicated that autologous and allogeneic MSC infusion is safe (Wang et al., 2021). Transient fever, administration site adverse events, sleeplessness, and constipation are the main adverse events; the study uncovered no serious safety effects. Fever is the most evident adverse effect, which may be caused by the immunomodulation function of MSCs and should be disclosed to patients before infusions. However, the long-term effects, such as tumorigenesis and emboli formation, remain to be investigated. Second, there is no standard guideline to ensure the quality of MSCs transplanted into the patients. MSCs from different tissues, ages, genders, and disease statuses may demonstrate varying characteristics and the prolonged *in vitro* culture expansion may induce senescence that impairs MSC differentiation and immunomodulatory functions. Therefore, there is an urgent need to establish a uniform standard. Another fundamental concern involves the obscurity of the relationship between the infusion program and the *in vivo* fate of MSCs. The top priority of most clinical trials is to evaluate the efficiency of MSCs, rendering it difficult to assess the optimal infusion dosage, frequency, and approach and the *in vivo* tracking of MSCs. A study on mice with acute liver injury optimized the MSC dosage and route (Li et al., 2015) and indicated that the superior mesenteric vein (SMV) was the optimal route for MSC infusion in liver disease, as MSCs were distributed widely in the liver and remained for 7 days post-transplantation, while MSCs were mostly trapped in the lungs after administration *via* the inferior vena cava (IVC) and resided in the injection region after intrahepatic (IH) injection. The authors also reported that the optimal delivery dose through the SMV was 2.5 × 10^5^ MSCs while a high dose of 0.5–1.0 × 10^6^ MSCs was followed by a high incidence of lethal portal vein embolization. In clinical trials, a single intravenous injection of MSCs is the most commonly applied program. In patients with liver cirrhosis, the minimum effective dose was 1 × 10^7^ MSCs, which lasted for 6 months with no adverse effects (Amin et al., 2013). Another study recorded no obvious difference between two infusions 1 month apart, which may have been caused by the short interval between the first and second injection (Suk et al., 2016). Therefore, the optimal infusion program still warrants future exploration to improve the therapeutic efficiency of MSCs. Last, for chronic liver diseases such as PBC, the disease stage at which MSCs should be applied remains to be investigated. Although there is much work that needs to be performed in this field, we found little improvement in the liver histology after MSC transplantation, as small interlobular bile duct fibrosis and cirrhosis are irreversible processes (Wang et al., 2014). Therefore, we speculated that MSC intervention at an early stage may be more effective.

On the other hand, no definitive perfect animal model can recapitulate human PBC pathogenesis, which limits in-depth mechanistic studies. The pathogenesis of PBC is complex and involves multiple factors and processes; therefore, ideal animal models would be valuable for clarifying PBC pathogenesis. There are currently two types of PBC mouse models (Katsumi et al., 2015): spontaneous models induced by genetic modification (e.g., NOD. c3c4 mice and dominant-negative TGF-β receptor II mice) and models induced by chemical xenobiotics and microbial immunization (e.g., 2OA-BSA-immunized mice and *Escherichia coli*–infected mice). These animal models can simulate the serological, immunological, and histopathological aspects of PBC. However, PBC progression is greatly accelerated in these models than that in humans, as PBC is a middle-age-onset disease, while animals demonstrate different responses to chemicals or microbials and extrapolation is difficult. Therefore, no single animal model can fully illustrate the pathogenesis of human PBC and there is a pressing need to explore new animal models that can mimic the slow PBC occurrence in the future.

Increasing attempts have been made to improve MSC treatment efficacy, which could facilitate future MSC applications for treating PBC. Priming MSCs with proinflammatory factors (e.g., IFN-γ, IL-1α, and IL-1β), hypoxia, and 3D culture materials boost MSC survival, function, and therapeutic effects (Zhou et al., 2021). Genetically modified MSCs with specific gene expression such as that for *CCL2* have demonstrated improved therapeutic potential in brain repair (Lee et al., 2020). Furthermore, MSC EVs exhibit valuable clinical importance in patients with autoimmune disease (Shen et al., 2021), refractory GVHD (Lai et al., 2018), and Alzheimer’s disease (Guo et al., 2020), which suggests promising means of treating PBC. Moreover, with the advent of somatic reprogramming technology, induced pluripotent stem cells can differentiate into MSCs (iMSCs) and can serve as an infinite source of iMSCs. iMSCs have been demonstrated to meet the qualities and function of MSCs (Zhao and Ikeya, 2018) and exert regenerative, reparative, and immunomodulatory effects in animal models of periodontal defection (Hynes et al., 2013), myocardial infarction (Liang et al., 2017), and inflammatory bowel disease (Soontararak et al., 2018). The development of iMSCs and their combination with new technologies such as gene editing and 3D culture present new strategies for treating PBC.
